# On fractional numerical simulation of HIV infection for CD8^+^ T-cells and its treatment

**DOI:** 10.1371/journal.pone.0265627

**Published:** 2022-03-24

**Authors:** R. A. Alharbey, Noufe H. Aljahdaly

**Affiliations:** 1 Mathematics Department, Faculty of Science, Al-Sulymania Women’s Campus, King AbdulAziz University, Jeddah, Kingdom of Saudi Arabia; 2 Mathematics Department, Faculty of Sciences and Arts, King Abdulaziz University, Rabigh, Saudi Arabia; Cardiff University, UNITED KINGDOM

## Abstract

The AIDS is a chronic disease and the researchers still exert their high efforts to reach the cure of HIV infection. The most common treatment is the antiretroviral therapy (cART) and the virus can be more effected if the patients stop using cART. The other problem is that the CD8+ T cells might be exhausted by persistent immune activation by cART. This paper introduces fractional-order into a mathematical model of HIV infection combining with stem cell therapy and control the infection by the immune system cells (CD8+ T cells). The paper introduced the numerical solutions for the mathematical model. The results show that the stem cell therapy with the activation of immune system cells might causes the cure for a HIV patient. This results are consistent with medical studies. Also, we proposed the effect of the fractional order (*α*) on the figures of the components.

## Introduction

Human Immunodeficiency Virus (HIV) is one of the most dangerous viruses in the world. Up to now, the infection by HIV virus is very hard to cure. The virus has killed over 25 million people since 1980 [1]. Since then researches and scientists put high efforts to analyze the mechanism of the virus to reach the optimal treatments such as antiretroviral therapy [2] or chemotherapy [3]. The initial stage of the infection starts by increasing the viral replication highly up to six weeks. Next stage is actually asymptomatic stage and has highly immune response and continue for several years. If the patients are not treated in this stage, the virus might convert to AIDS disease. However, the HIV virus attacks the CD4^+^ T-cells because these cells have protein on their surface whose ability to bind to foreign substances such viruses. Thus, the CD4^+^ T-cell (*T*) is converted to DNA once it is effected by the virus. Then, the virus multiplies inside the cells rapidly. The thymus is triggered to produce more CD4^+^ T-cells and then more viruses. Consequently, the CD4^+^ T lymphocytes are destructed, the immune system loss its power and the helper of the cells that help to build a robust immune response is damaged [4].

The most common treatment for AIDS is the combined antiretroviral therapy (cART) which improve the immune reconstitution. It is used as pre-exposure or post-exposure prophylaxis and as vaccine to prevent the transmission. The cART makes the HIV infection as chronic disease and under control clinically. Even though, the cART alone can not end the epidemic because the virus transmission is increase rapidly when the individual stops using it due to rebound from viral reservoirs during cART usage [5]. However, some researches indicate that the infected individuals are able to control the disease progresses by effective HIV-specific CD8+ T cells and without using the cART. There is CD8+ T cell-mediated mechanism of durable HIV control. Thus, the CD8^+^ T cells are able to limit the transmission of viruses. The other treatment is stem cell but it does not used widely yet because of its cost and limitation of suitable donors [6].

Therefore, the mathematical model is one of the initial study to predict the results of available treatments before starting clinical experiments [7]. The HIV infection has been described by mathematical model [8] including three components in individuals’ blood: (i) concentration of unaffected CD4^+^ T-cells, (ii) concentration of affected CD4^+^ T-cells and (iii) concentration of virus [9, 10]. Some researchers studied the treatment of HIV infection by stem cell [11] or by investigating the effect of the CD8^+^ T cells on the HIV-1 virus [12].

However, the novelty on this paper is investigating the effect of both CD8+ T cells as well as stem cell transplants on HIV-1 virus by study the dynamic of the numerical solutions. The associated HIV-1 model is investigated computationally and numerically with the aid of fractional derivative equations of order *α*, where 0 < *α* < 1, is a memory index order of fractional differential equations (FDE). It is a promising approach due its ability for describing memory phenomena [13]. Types of FDE involving Riemann-Liouville sequential fractional derivative, Caputo’s definition [14].

This paper is organized as follow: in section (2), the fractional mathematical model of HIV infection account to CD8+ T cells, cART and stem cells (SCs), in section (3), the numerical simulation of the considered model using Caputo’s definition of fractional derivative of order *α*, section (4), is summarized the results.

## The HIV model equations

### Ordinary derivative case

The HIV model provides a good example for understanding the dynamics of in-vivo interaction of HIV and the immune system cells. The HIV model in reference [15] is modified by adding the effect of stem cell therapy. Therefore, the modified model is constructed into six components. These variables are: *S*(*t*) is the concentration of SCs. The healthy (uninfected) and the infected CD4^+^ T cells are denoted by *T*(*t*), *I*(*t*), respectively. *V*(*t*) is the concentration of HIV virus. *Z*(*t*) is the immune system cells (CD8^+^ T cells). *Z*_*a*_(*t*) is the activated immune system cells. The interaction variables and parameters are summarized in Table 1. In addition, SCs are divided by the rate *k*. The probability of the type of SCs’ division are: (i) division into two undifferentiated cells at rate *α*_*s*_, (ii) division into undifferentiated cell and differentiated cell at rate *α*_*D*_ and (iii) division into two differentiated cells at rate *α*_*T*_ such that *α*_*A*_ + *α*_*s*_ + *α*_*D*_ = 1 [13, 16]. Since the system in fractional calculus is more accurate, we will consider the fractional system where all the parameters are depended on *α* [17–19]. The fractional mathematical nonlinear HIV model is given by,

$$\begin{array}{cc} \frac{d^{\alpha }S\left(t\right)}{dt^{\alpha }} & =(k^{\alpha }\left(\alpha_{s}^{\alpha }-\alpha_{D}^{\alpha }\right)-\mu_{S}^{\alpha })S\left(t\right)=F_{1}\left(t,S\left(t\right),T\left(t\right),I\left(t\right),V\left(t\right),Z\left(t\right),Z_{a}\left(t\right)\right)\\ \frac{d^{\alpha }T\left(t\right)}{dt^{\alpha }} & =\lambda_{T}^{\alpha }+\left(2\alpha_{D}^{\alpha }+\alpha_{A}^{\alpha }\right)k^{\alpha }A^{\alpha }S\left(t\right)-\mu_{T}^{\alpha }T\left(t\right)-K_{T}^{\alpha }T\left(t\right)V\left(t\right)\\ & =F_{2}\left(t,S\left(t\right),T\left(t\right),I\left(t\right),V\left(t\right),Z\left(t\right),Z_{a}\left(t\right)\right)\\ \frac{d^{\alpha }I\left(t\right)}{dt^{\alpha }} & =K_{T}^{\alpha }T\left(t\right)V\left(t\right)-\mu_{I}^{\alpha }I\left(t\right)-\gamma^{\alpha }I\left(t\right)Z_{A}\left(t\right)=F_{3}\left(t,S\left(t\right),T\left(t\right),I\left(t\right),V\left(t\right),Z\left(t\right),Z_{a}\left(t\right)\right)\\ \frac{d^{\alpha }V\left(t\right)}{dt^{\alpha }} & =N\mu_{I}^{\alpha }I\left(t\right)-\mu_{V}^{\alpha }V\left(t\right)=F_{4}\left(t,S\left(t\right),T\left(t\right),I\left(t\right),V\left(t\right),Z\left(t\right),Z_{a}\left(t\right)\right)\\ \frac{d^{\alpha }Z\left(t\right)}{dt^{\alpha }} & =\lambda_{Z}^{\alpha }+\left(2\alpha_{D}^{\alpha }+\alpha_{A}^{\alpha }\right)k^{\alpha }B^{\alpha }S\left(t\right)-\mu_{Z}^{\alpha }Z\left(t\right)-\beta^{\alpha }Z\left(t\right)I\left(t\right)\\ & =F_{5}\left(t,S\left(t\right),T\left(t\right),I\left(t\right),V\left(t\right),Z\left(t\right),Z_{a}\left(t\right)\right)\\ \frac{d^{\alpha }Z_{A}\left(t\right)}{dt^{\alpha }} & =\beta^{\alpha }Z\left(t\right)I\left(t\right)-\mu_{Z_{A}}^{\alpha }Z_{A}\left(t\right)=F_{6}\left(t,S\left(t\right),T\left(t\right),I\left(t\right),V\left(t\right),Z\left(t\right),Z_{a}\left(t\right)\right). \end{array}$$ (1)

The theoretical study for the model is similar to the model in reference [15]. The importance of our study is finding the solutions for the modified system with connecting the study with the biological respective. The basic reproduction number is $R_{0}=\left(NK_{T}^{\alpha }\lambda_{T}^{\alpha }\right)/\left(\mu_{T}^{\alpha }\mu_{V}^{\alpha }\right)$ which indicates to the secondary infection by single virus in *T*–cell and it measures the virus spread in patient body. Therefore, the free virus equilibrium point is local stable if *R*_0_ < 1, and is unstable if *R*_0_ ≥ 1. However, if *R*_0_ > 1 indicates the large disease epidemic. Consequently, it is important to let 0 < *R*_0_ < 1 to control virus spread.

**Table 1:** The definition of the variables in system (4).

variable	meaning
*K*_*T*_	rate of infection T-cells
λ_*T*_	rate of producing *T*-cell in bone marrow and thymus
λ_*Z*_	rate of producing *Z*-cell
*μ*_*T*_	rate of decaying for susceptible *T*
*r*_*T*_	rate of *T* mitosis
*μ*_*S*_	natural death rate of *S*
*μ*_*I*_	natural death rate of *I*
*μ*_*V*_	the death rate of *V*
*μ*_*Z*_	the death rate of *Z*
$\mu_{Z_{A}}$	rate of decaying for *Z*_*A*_
*γ*	rate of eliminating the *I* cells by *Z*_*A*_
*N*	the number of virus particle produced by each *I*-cell
*β*	rate of activation of *Z* due to the attendance *I*-cells.
*A*	Amplification factor
*B*	Amplification factor

### Fractional derivative approach

In this approach, we adopt the Caputo’s *n*^*th*^ order fractional derivative [20],

$$\begin{array}{c} \frac{d^{\alpha }}{dr^{\alpha }}f^{\left(n\right)}\left(t\right)=\frac{1}{\Gamma (n-\alpha )}\int_{0}^{t}\frac{1}{{\left(t-t^{\prime }\right)}^{\left(1-n+\alpha \right)}}\;f^{\left(n\right)}\left(t^{\prime }\right)\;dt^{\prime };\;n=1,2,\ldots . \end{array}$$ (2)

where Γ(*x*) is the Gamma function.

The 1^*st*^ order derivative $Q^{\prime }\left(t\right)=\left\{S^{\prime }\left(t\right),T^{\prime }\left(t\right),I^{\prime }\left(t\right),V^{\prime }\left(t\right),Z^{\prime }\left(t\right),Z_{a}^{\prime }\left(t\right)\right\}$ term in Eq (1) becomes,

$$\begin{array}{c} Q^{\prime }\left(t\right)\to \frac{1}{\Gamma (1-\alpha )}\int_{0}^{t}\frac{1}{{\left(t-t^{\prime }\right)}^{\left(\alpha \right)}}\;Q^{\prime }\left(t^{\prime }\right)\;dt^{\prime }, \end{array}$$ (3)

so, within the Caputo’s fractional derivatives approach, the original ODE, Eq (1), with the use of Eq (3), transforms to integro-differential equation,

$$\begin{array}{c} \frac{d^{\alpha }Q\left(t\right)}{dt^{\alpha }}=\frac{1}{\Gamma (1-\alpha )}\int_{0}^{t}\frac{1}{{\left(t-t^{\prime }\right)}^{\alpha }}Q^{\prime }\left(t^{\prime }\right)dt^{\prime }=F, \end{array}$$ (4)

where, *F* = *F*_*i*_(*t*, *S*(*t*), *T*(*t*), *I*(*t*), *V*(*t*), *Z*(*t*), *Z*_*a*_(*t*)), *i* = 1, 2, 3, 4, 5, 6. The equation of stem cell is only in *S*(*t*), thus, we able to find the exact solution for the stem cell function.

## Numerical simulation and discussion

In literature, there are many powerful methods to solve the ODE numerically such as Adomian decomposition method [21, 22], Multistage differential transformation method [23, 24], the modified *G*′/*G*^2^ expansion method [25, 26], Tanh-expansion method [27], exponential time differencing method [28, 29], the generalized auxiliary equation Method [30], the 4th order Runge Kutta (RK4) method [31] or novel analytical methods [32]. Most of these methods are modified to work for fractional equations. In this paper, we will use the numerical technique of the Euler’s method to solve Eq (4) with the same ICs. as in Table 1 [33]. The system of Eq (4) subjects to the following initial conditions

$$S\left(0\right)=S_{o},\;\;T\left(0\right)=T_{o},\;\;T_{i}\left(0\right)=I_{o},\;\;V\left(0\right)=V_{o}.\;\;Z\left(0\right)=Z_{o},\;\;,Z_{a}\left(0\right)=Z_{a_{o}}$$

**Fig 1:**
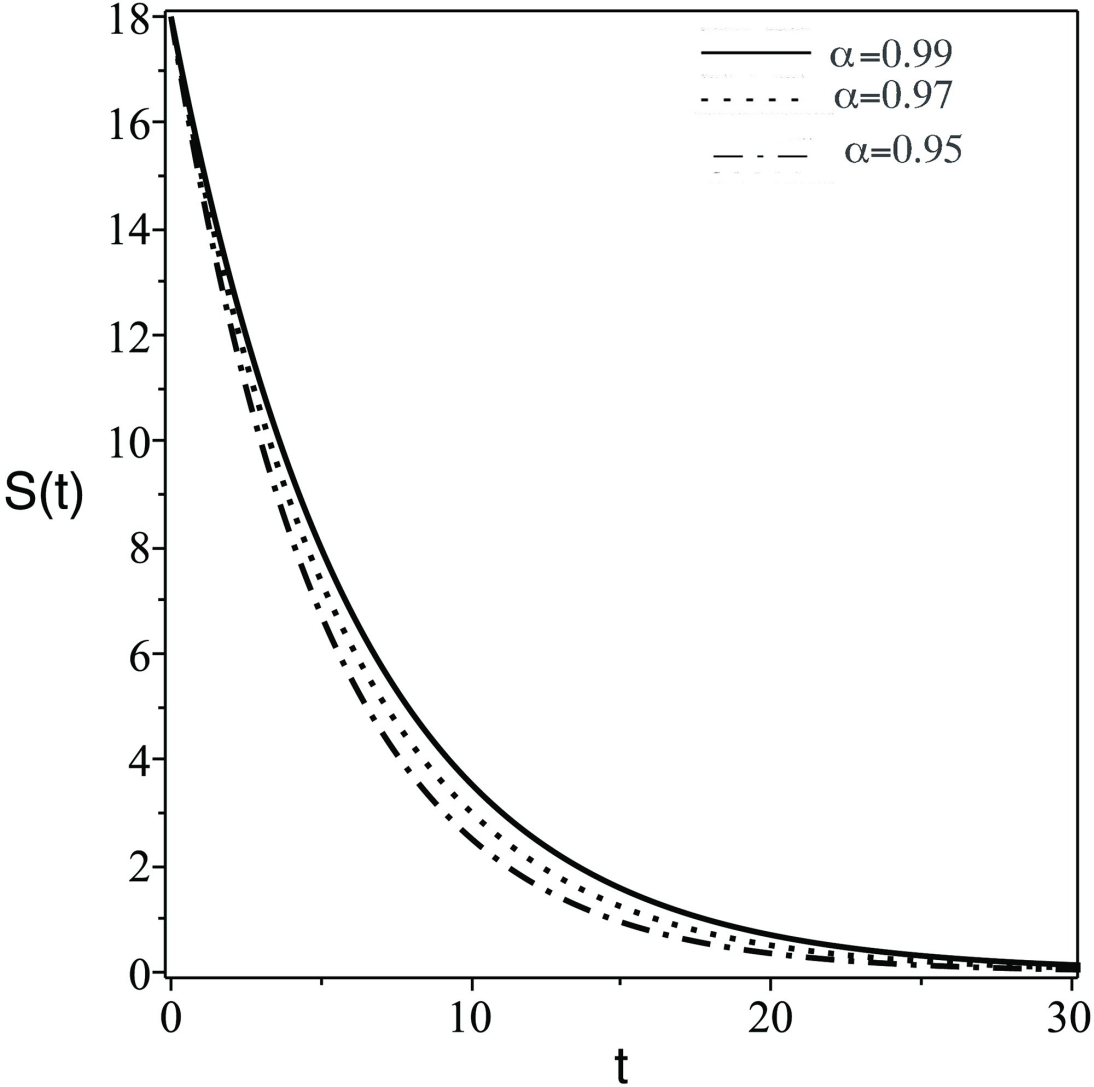
Number of the concentration of stem cells, *S*(*t*), against days (t) for different values of *α*.

**Fig 2:**
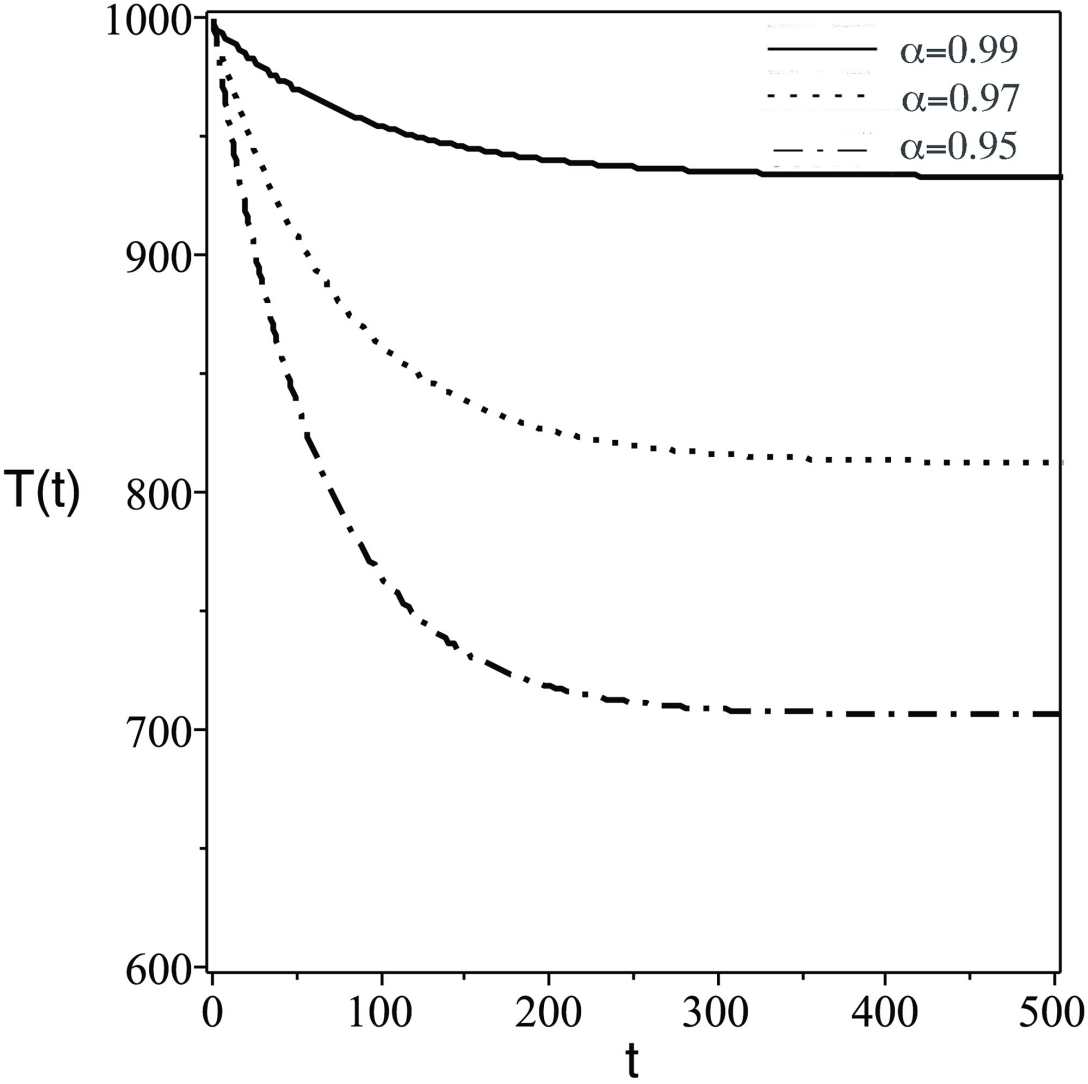
Same as Fig 1 but for the number of concentration of uninfected CD4^+^ T-cells, *T*(*t*).

**Fig 3:**
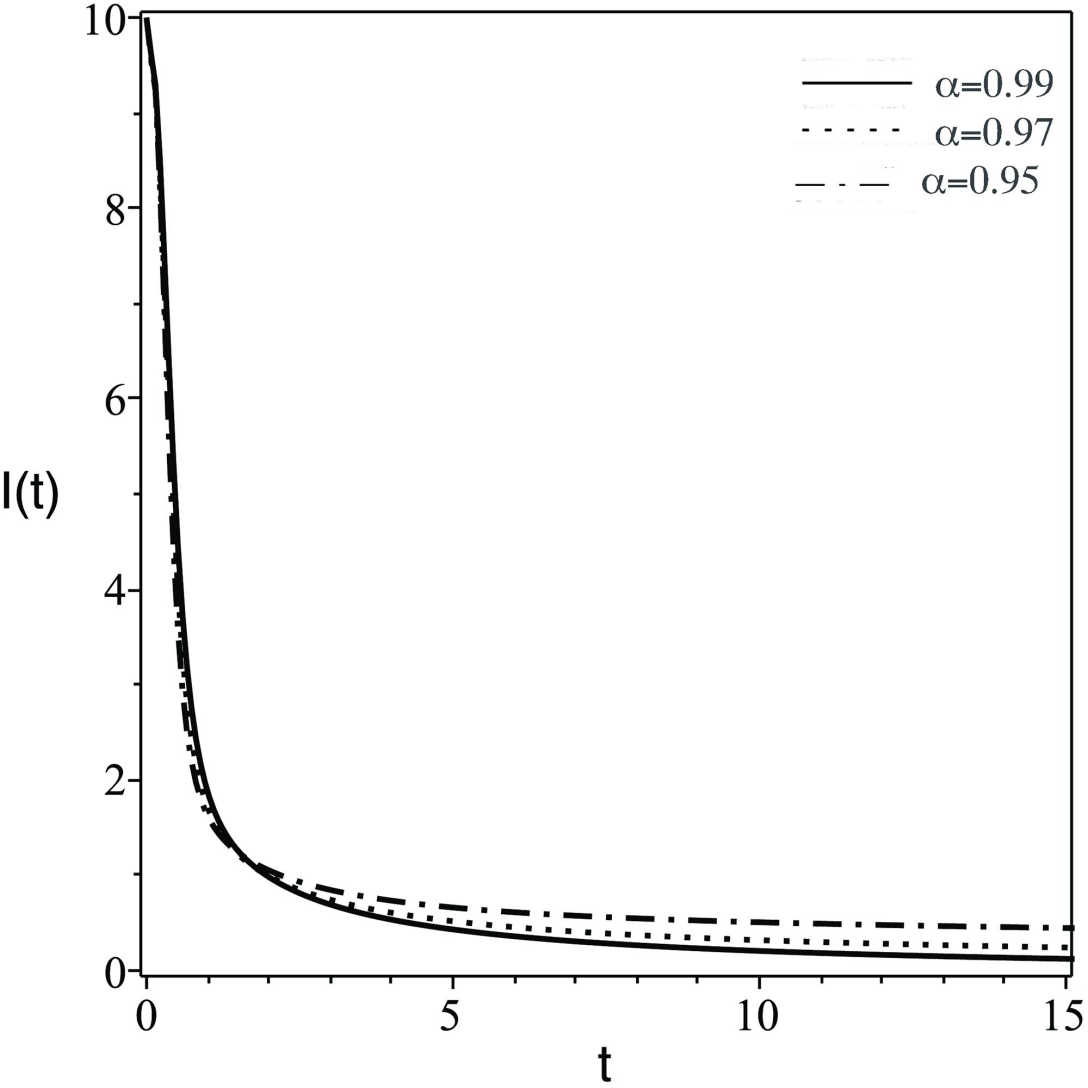
Same as Fig 1 but for the number of concentration of infected CD4^+^ T-cells, *I*(*t*).

**Fig 4:**
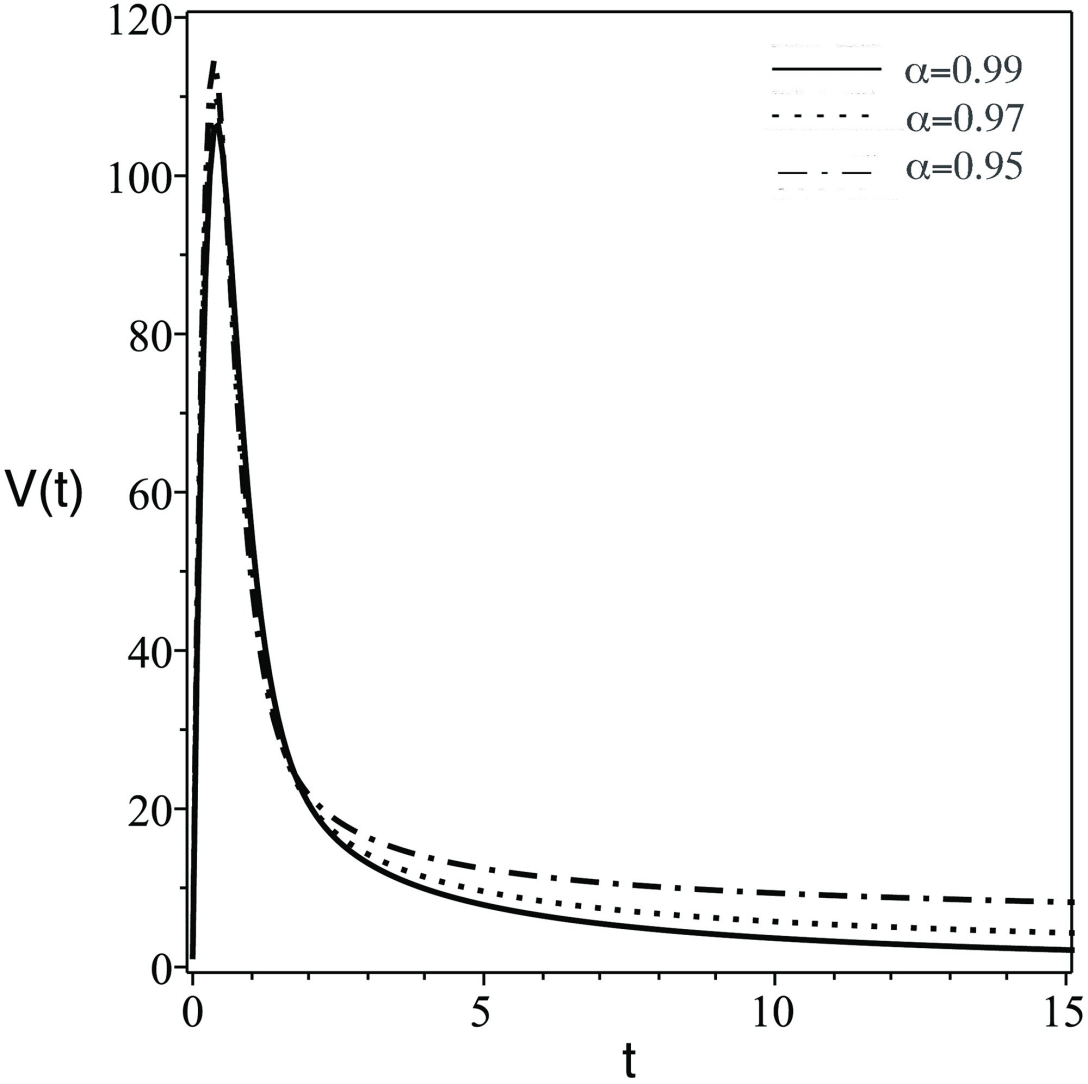
Same as Fig 1 but for the number of HIV virus in the blood, *V*(*t*).

The iterative numerical scheme can be described as follows,

(i) The initial values *t*(0) = *t*_*o*_ = 0, *S*(0) = 18, *T*(0) = 1000, *I*(0) = 10, *V*(0) = 1, *Z*(0) = 500 and $Z_{a_{o}}\left(0\right)=0$ and the interaction parameters are set (see Table 2).
(ii) The integro-diferential equations are obtained from the transformation of Caputo’s Eq (4) over the interval *t*(*days*) ∈ [0, *a*].
(iii) *S*(*t*_*j*_), *T*(*t*_*j*_), *I*(*t*_*j*_), *V*(*t*_*j*_) *Z*(*t*_*j*_) *and* *Z*_*a*_(*t*_*j*_) are generated with fractional Euler’s method approximation scheme,
  $$\begin{array}{c} Q\left(t_{j}\right)=Q\left(t_{j-1}\right)+d\frac{h^{\alpha }}{\Gamma (\alpha +1)}F_{i}\left(t_{j-1}\right), \end{array}$$ (5)
  where, 0 ≤ *j* ≤ *n*, *t*_*j*_ = *t*_*o*_ + *jh* and *h* = (*a* − *t*_*o*_)/*η* is the step size, *η* is the iteration number.
(iv) A set of points, (*t*_*j*_, *Q*(*t*_*j*_)), is produced for different values of *α*.
(v) According to (iv), Figs 1–4 are obtained.

**Table 2:** The value of the parameters based on a realistic analysis [34].

Parameter	Value	Parameter	Value
λ_*T*_	0.17 cells/ul.day	*r*_*T*_	3
λ_*Z*_	20 cell/mm3/day	*α*_*S*_	0.24/day
*μ*_*T*_	0.01 day^−1^	*K*_*T*_	0.03/day
*μ*_*I*_	0.5 day^−1^	*N*	100 vir. cell^−1^ day^−1^
*μ*_*V*_	3 day^−1^	*μ*_*S*_	0.17 cells/ul.day
*μ*_*Z*_	0.06 day^−1^	*γ*	0.02 day^−1^
$\mu_{Z_{A}}$	0.004 day^−1^	*β*	0.004 day^−1^
*T*_*o*_	100 cells/ul	*Z*_*o*_	500
*I*_*o*_	10 cells/ul	$Z_{A_{o}}$	0
*V*_*o*_	1 virus/ul	*S*_0_	18 cells/ul
*S*_*o*_	18	*α*_*D*_	0.16/day
*μ*_*S*_	0.03/day	*A*	0.5
*α*_*A*_	0.6 cells/ul.day	*B*	0.25

**Fig 5:**
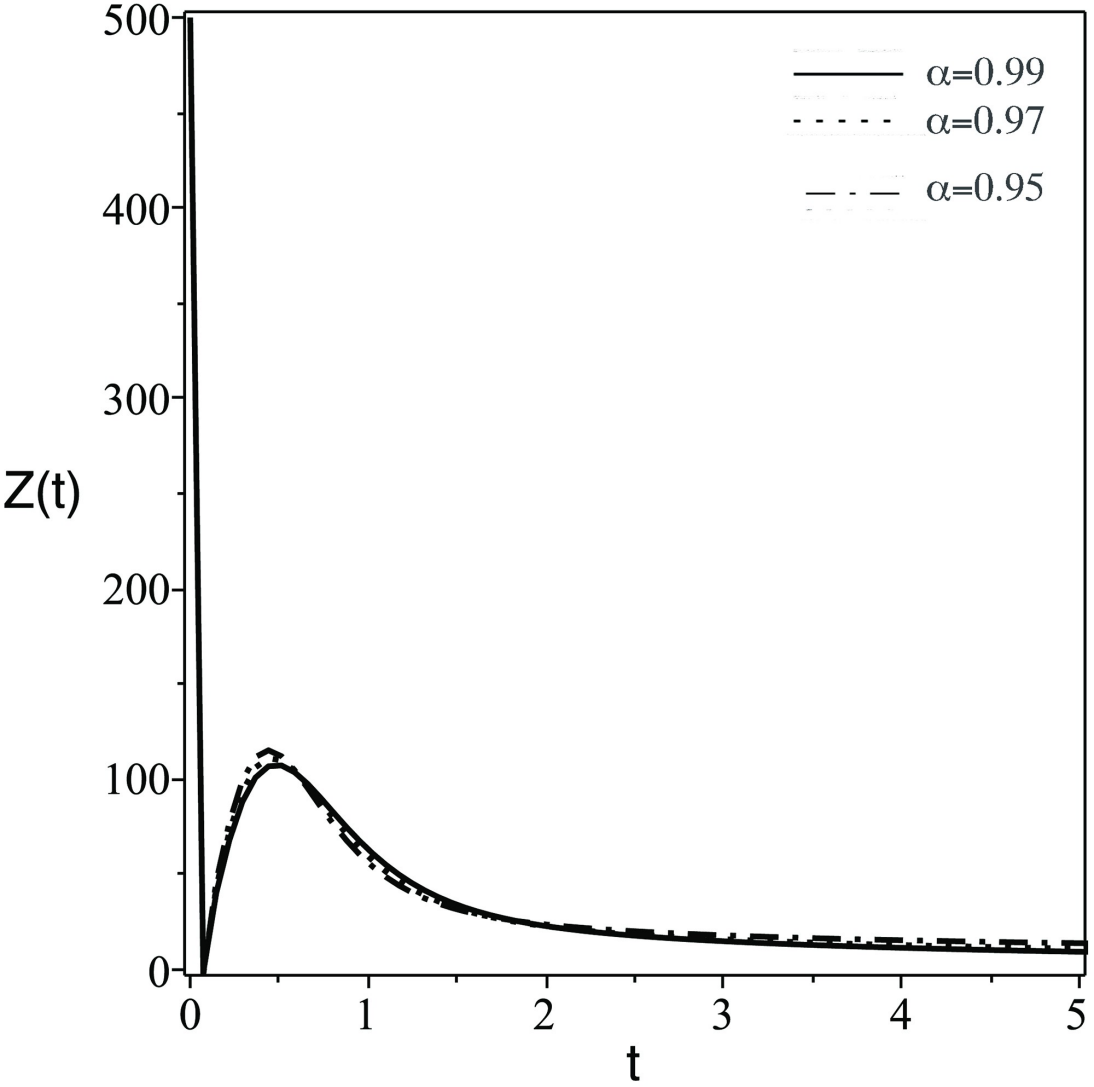
Same as Fig 1 but for the number of immune system cells (CD8^+^ T cells), *Z*(*t*).

**Fig 6:**
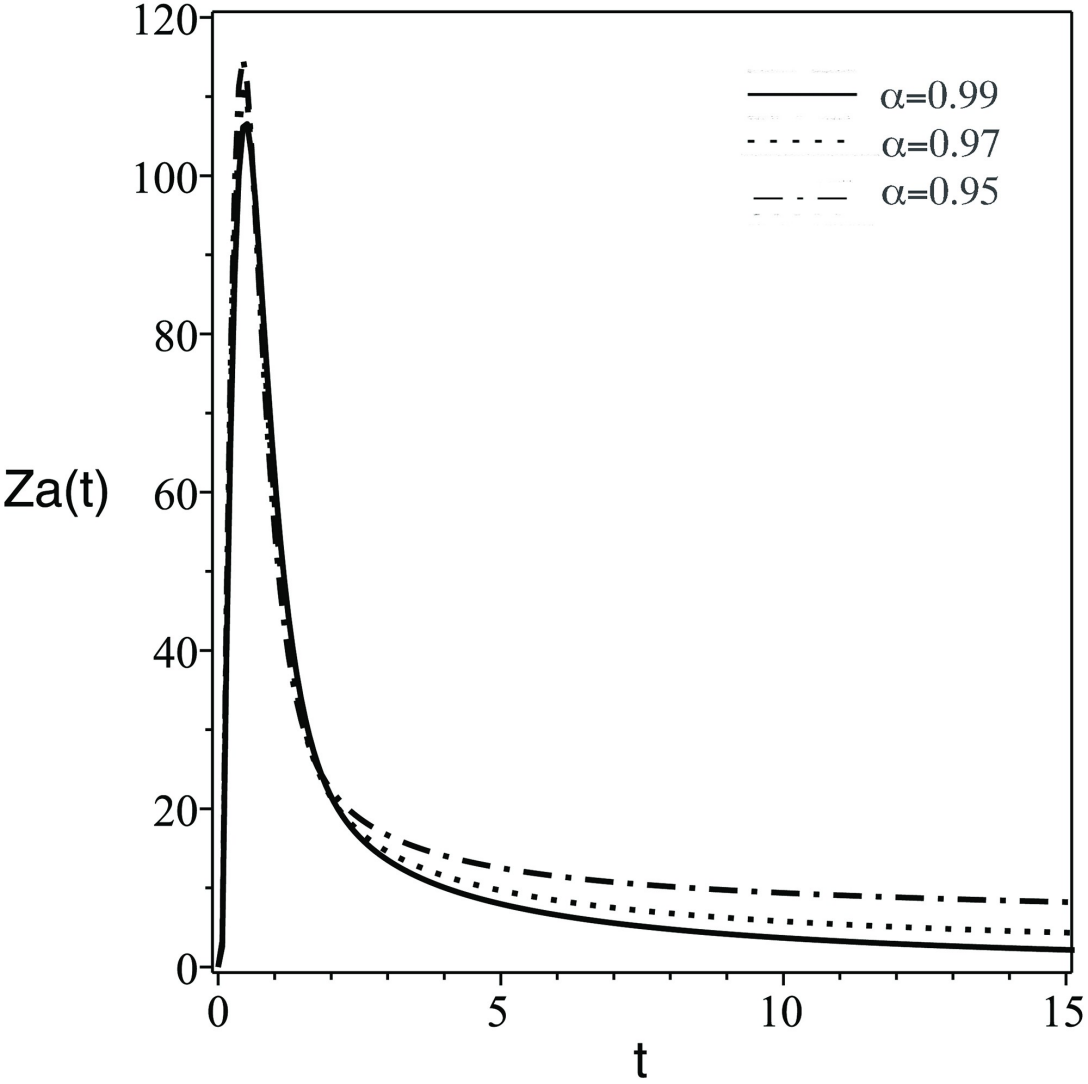
Same as Fig 1 but for the number of activated immune system cells, *Z*_*a*_(*t*).

The plots of the six components *Q*(*t*) = {*S*(*t*), *T*(*t*), *I*(*t*), *V*(*t*), *Z*(*t*), *Z*_*a*_(*t*)} against *t* (days) for different values of FDE order (*α*) are displayed. The numerical solutions of the system predict the dynamic of the model components as follows:

- In Fig 1, the number of the stem cells in the blood decreases dramatically. Clinically speaking, stem cells enhance the growth of healthy cells and differentiation. Therefore, the transplanted stem cell differentiate into body cells or another stem cells.
- In Fig 2, the concentration of uninfected CD4^+^ T-cells, *T*(*t*) increase due to the effect of stem cells which generate healthy body cells.
- In Fig 3, the concentration of infected CD4^+^ T-cells, *T*(*t*) decrease due to the effect the treatments
- In Fig 4, the HIV virus decrease rapidly specially for the case of smaller *α*, this is due to the effect of immune system cells and the treatments.
- In Fig 5, huge number of CD8^+^ T cells are produced due to the present of virus, then the concentration of CD8^+^ T cells decrease after the virus and infected cell fade away in the body.
- In Fig 6, activated CD8^+^ T cells increase highly on the beginning of infection to attack the virus and infected cell, after that the concentration of *Z*_*a*_ return to normal level.
- The small order of fractional derivative (*α*) indicates more effect on components of the system.

The novelty of this study, that the numerical solutions predict that the cure of HIV-1 infection might be reach by control the disease progresses by effective HIV-specific CD8+ T cells combining with the stem cell therapy. In literature, there are two medical cases showed the cure of HIV infection. First one is the Berlin Patient who got cure after stem cell transplant from a homozygous donor. Second case is the London patient who got stem-cell transplantation [35]. However, we realized from our previous numerical study [13] for the fractional mathematical model of HIV-1 with only stem cell treatment that the treatment with only stem cell therapy might increase the quality of patient’s life for short time, but does not reach the cure. This result is agree with two medical cases in references [5, 11].

## Conclusion

In this study, we suggested a new model of interaction of an in-vivo HIV in the presence of CD8^+^ T cells and stem cells. The studied mathematical model predicts that after the stem cell transplants and control the disease progresses by effective HIV-specific CD8+ T cells the patient might can be cured. The numerical solutions showed the increasing of the *T*- cells, decreasing *V* and *I* cells and enhancing the *Z* and *Z*_*a*_ cells. In general the numerical solutions are consistent with the medical cases in literature which showed the cure of HIV infection in two patients while two case studies showed that the stem cell therapy alone can only improve the quality life of patients for short period [5, 11].

Finally, we use the definition of the fractional derivatives which is more convenient by using Caputo’s definition. It might be useful as well by adopting Riemann-Liouville definition. This will be presented as a future work. In addition, we aim to study in the future the effect of diffusion of virus on the blood where the system of ordinary differential equations will transfer to a system of partial differential equations.
